# 2-Methyl­sulfanyl-5,6-dihydro-2*H*-1,3-dithiolo[4,5-*b*][1,4]dioxin-2-ium tetra­fluoro­borate

**DOI:** 10.1107/S1600536812005326

**Published:** 2012-03-03

**Authors:** Guoquan Zhou, Xinzhi Chen

**Affiliations:** a Department of Chemical and Biological Engineering, Zhejiang University, Hangzhou 310027, People’s Republic of China; b College of Chemical Engineering, Ningbo University of Technology, Ningbo 315016, People’s Republic of China

## Abstract

The title compound, C_6_H_7_O_2_S_3_^+^·BF_4_^−^, consists of a planar 2-thioxo-1,3-dithiol-4,5-yl unit [maximum deviation from the ring plane = 0.020 (3) Å], with an ethyl­enedi­oxy group fused at the 4,5-positions; the ethyl­enedi­oxy C atoms are disordered over two positions with site-occupancy factors of 0.5. The 1,4-dioxine ring has a twist-chair conformation. Weak cation–anion S⋯F inter­actions [3.022 (4)–3.095 (4) Å] and an S⋯O [3.247 (4) Å] inter­action are present.

## Related literature

For background on metal–organic coordination compounds, see: Chen *et al.* (2000 ▶); Xiong *et al.* (1999 ▶). For the preparation and crystal structure of a related compound, see: Han & Zhang (2010 ▶); Kanchanadevi *et al.* (2010 ▶).
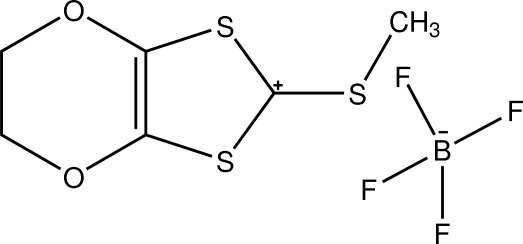


## Experimental

### 

#### Crystal data

C_6_H_7_O_2_S_3_^+^·BF_4_^−^
*M* *_r_* = 294.14
Monoclinic, 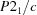

*a* = 10.7410 (13) Å
*b* = 10.1175 (10) Å
*c* = 10.1874 (11) Å
β = 94.488 (4)°
*V* = 1103.7 (2) Å^3^
*Z* = 4
Mo *K*α radiation
μ = 0.71 mm^−1^
*T* = 223 K
0.40 × 0.35 × 0.35 mm

#### Data collection

Rigaku Saturn CCD diffractometer
Absorption correction: multi-scan (*REQAB*; Jacobson, 1998 ▶) *T*_min_ = 0.593, *T*_max_ = 0.781
6014 measured reflections
2046 independent reflections
1749 reflections with *I* > 2σ(*I*)
*R*_int_ = 0.026

#### Refinement

*R*[*F* ^2^ > 2σ(*F* ^2^)] = 0.063
*wR*(*F* ^2^) = 0.174
*S* = 1.07
2046 reflections
147 parameters
6 restraints
H-atom parameters constrained
Δρ_max_ = 0.69 e Å^−3^
Δρ_min_ = −0.55 e Å^−3^

### 

Data collection: *CrystalClear* (Rigaku, 2005 ▶); cell refinement: *CrystalClear*; data reduction: *CrystalStructure* (Rigaku/MSC, 2005 ▶); program(s) used to solve structure: *SHELXS97* (Sheldrick, 2008 ▶); program(s) used to refine structure: *SHELXL97* (Sheldrick, 2008 ▶); molecular graphics: *SHELXTL* (Sheldrick, 2008 ▶); software used to prepare material for publication: *SHELXTL*.

## Supplementary Material

Crystal structure: contains datablock(s) I, global. DOI: 10.1107/S1600536812005326/rk2328sup1.cif

Structure factors: contains datablock(s) I. DOI: 10.1107/S1600536812005326/rk2328Isup2.hkl

Supplementary material file. DOI: 10.1107/S1600536812005326/rk2328Isup3.cml

Additional supplementary materials:  crystallographic information; 3D view; checkCIF report

## Figures and Tables

**Fig. 1.**
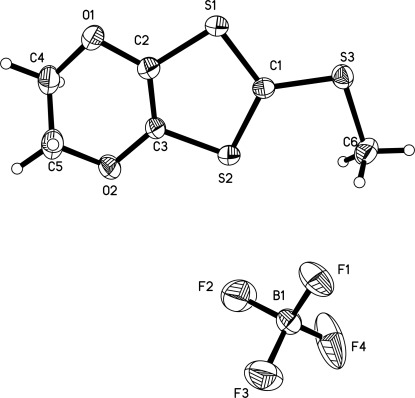
The molecular structure of the title compound with the atom numbering scheme. Displacement ellipoids are drawn at the 30% probability level. H atoms are presented as a small spheres of arbitrary radius. Only initial disordered C atoms are presented.

**Fig. 2.**
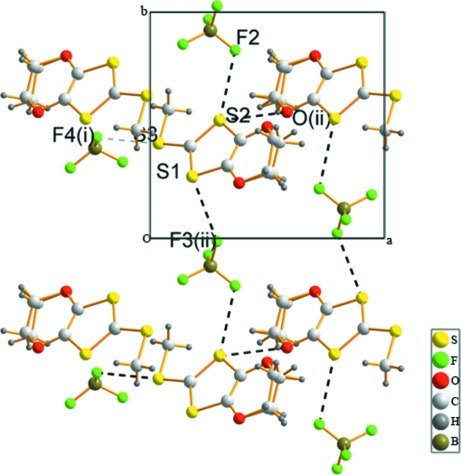
The crystal packing diagram view along c axis. Dashed lines indicate the weak interaction. Symmetry codes: (i) -x, -1/2+y, 1/2-z; (ii) x, -1+y, z; (iii) 1-x, 1-y, 1-z.

## supplementary crystallographic information

### Comment

The construction of metal-organic coordination compounds has attracted much attention owing to potential functions, such as permittivity, fluorescence, magnetism and optical properties (Chen *et al.*, 2000; Xiong *et al.*, 1999). We report here the molecular and crystal structures of the title compound, 5,6-dihydro-2-(methylthio)-[1,3]dithiolo[4,5-*b*][1,4]dioxine tetrafluoroborate, I. In I, (Fig. 1), the inductive effects of the oxygen atoms makes the between C4 and C5 bond length longer (1.493Å). The C–S bond lengths range 1.681 (4)Å-1.723 (5)Å is small than that typical of C–S bond lengths, 1.82Å, suggesting a degree of conjugation in the dithiol-2-thione system. Both conformers of the disordered dioxane ring adopt half-chair conformations. In I, there are weak S···F interaction (3.095 (4)Å-3.022 (4)Å] and S···O (3.247 (4)Å). These interaction (cation-anion) in the crystal structure, forming a one-dimensional network, see (Fig. 2).

### Experimental

The 5,6-dihydro-[1,3]dithiolo[4,5-*b*][1,4]dioxine-2-thione dissolve in acetonitrile with [(CH_3_)_3_O]^+^×[BF_4_]^-^ and get the yellow compound. Slow evaporation of the compound in a solution of *THF* gave single crystals suitable for *X*-ray analysis.

### Refinement

All the H atoms were placed in geometrically calculated positions, with C–H = 0.98Å (methylene) and 0.97Å (methyl) and *U*_iso_(H) = 1.2 *U*_eq_(C) (methylene) and 1.5 *U*_eq_(C) (methyl), and refined using a riding model.

### Crystal data

**Table:**

C_6_H_7_O_2_S_3_^+^·BF_4_^−^	*F*(000) = 592
*M**_r_* = 294.14	*D*_x_ = 1.770 Mg m^−^^3^
Monoclinic, *P*2_1_/*c*	Mo *K*α radiation, λ = 0.71075 Å
Hall symbol: -P 2ybc	Cell parameters from 2895 reflections
*a* = 10.7410 (13) Å	θ = 3.3–27.5°
*b* = 10.1175 (10) Å	µ = 0.71 mm^−^^1^
*c* = 10.1874 (11) Å	*T* = 223 K
β = 94.488 (4)°	Block, yellow
*V* = 1103.7 (2) Å^3^	0.40 × 0.35 × 0.35 mm
*Z* = 4	

### Data collection

**Table:**

Rigaku Saturn CCD diffractometer	2046 independent reflections
Radiation source: fine-focus sealed tube	1749 reflections with *I* > 2σ(*I*)
Graphite monochromator	*R*_int_ = 0.026
Detector resolution: 14.63 pixels mm^-1^	θ_max_ = 25.5°, θ_min_ = 3.3°
ω–scan	*h* = −12→13
Absorption correction: multi-scan (*REQAB*; Jacobson, 1998)	*k* = −10→12
*T*_min_ = 0.593, *T*_max_ = 0.781	*l* = −11→12
6014 measured reflections	

### Refinement

**Table:**

Refinement on *F*^2^	Primary atom site location: structure-invariant direct methods
Least-squares matrix: full	Secondary atom site location: difference Fourier map
*R*[*F*^2^ > 2σ(*F*^2^)] = 0.063	Hydrogen site location: inferred from neighbouring sites
*wR*(*F*^2^) = 0.174	H-atom parameters constrained
*S* = 1.07	*w* = 1/[σ^2^(*F*_o_^2^) + (0.0986*P*)^2^ + 1.5661*P*] where *P* = (*F*_o_^2^ + 2*F*_c_^2^)/3
2046 reflections	(Δ/σ)_max_ < 0.001
147 parameters	Δρ_max_ = 0.69 e Å^−^^3^
6 restraints	Δρ_min_ = −0.55 e Å^−^^3^

### Special details

**Table:**

Geometry. All s.u.'s (except the s.u. in the dihedral angle between two l.s. planes) are estimated using the full covariance matrix. The cell s.u.'s are taken into account individually in the estimation of s.u.'s in distances, angles and torsion angles; correlations between s.u.'s in cell parameters are only used when they are defined by crystal symmetry. An approximate (isotropic) treatment of cell s.u.'s is used for estimating s.u.'s involving l.s. planes.
Refinement. Refinement of *F*^2^ against ALL reflections. The weighted *R*-factor *wR* and goodness of fit *S* are based on *F*^2^, conventional *R*-factors *R* are based on *F*, with *F* set to zero for negative *F*^2^. The threshold expression of *F*^2^ > σ(*F*^2^) is used only for calculating *R*-factors(gt) *etc*. and is not relevant to the choice of reflections for refinement. *R*-factors based on *F*^2^ are statistically about twice as large as those based on *F*, and *R*-factors based on ALL data will be even larger.

### Fractional atomic coordinates and isotropic or equivalent isotropic displacement parameters (Å^2^)

**Table:**

	*x*	*y*	*z*	*U*_iso_*/*U*_eq_	Occ. (<1)
S1	0.15907 (11)	0.25827 (11)	0.56191 (12)	0.0482 (4)	
S2	0.26927 (10)	0.49599 (10)	0.46964 (11)	0.0428 (3)	
S3	0.02403 (10)	0.39889 (11)	0.34288 (10)	0.0439 (3)	
F1	0.1210 (3)	0.8199 (4)	0.4652 (3)	0.0815 (11)	
F2	0.3311 (4)	0.7944 (4)	0.4588 (5)	0.1015 (14)	
F3	0.2587 (4)	0.9828 (4)	0.5236 (5)	0.0953 (13)	
F4	0.2242 (5)	0.9251 (8)	0.3140 (4)	0.161 (3)	
O1	0.3475 (3)	0.2304 (3)	0.7435 (3)	0.0566 (9)	
O2	0.4604 (3)	0.4681 (3)	0.6479 (3)	0.0528 (8)	
C1	0.1508 (4)	0.3871 (4)	0.4559 (4)	0.0368 (9)	
C2	0.3004 (4)	0.3043 (4)	0.6403 (4)	0.0427 (10)	
C3	0.3528 (4)	0.4142 (4)	0.5960 (4)	0.0388 (9)	
C4	0.4786 (15)	0.2727 (17)	0.7661 (15)	0.083 (3)	0.50
H4A	0.5251	0.2454	0.6916	0.099*	0.50
H4B	0.5169	0.2303	0.8457	0.099*	0.50
C4'	0.4634 (16)	0.2771 (19)	0.8065 (15)	0.083 (3)	0.50
H4'1	0.5278	0.2114	0.7917	0.099*	0.50
H4'2	0.4548	0.2801	0.9015	0.099*	0.50
C5	0.486 (2)	0.4194 (17)	0.7811 (18)	0.083 (3)	0.50
H5A	0.4229	0.4514	0.8384	0.099*	0.50
H5B	0.5686	0.4469	0.8179	0.099*	0.50
C5'	0.509 (2)	0.4054 (18)	0.7666 (19)	0.083 (3)	0.50
H5'1	0.4964	0.4670	0.8387	0.099*	0.50
H5'2	0.5991	0.3969	0.7616	0.099*	0.50
C6	0.0632 (5)	0.5445 (5)	0.2538 (5)	0.0603 (13)	
H6A	0.0631	0.6207	0.3117	0.091*	
H6B	0.0021	0.5575	0.1797	0.091*	
H6C	0.1454	0.5340	0.2221	0.091*	
B1	0.2316 (5)	0.8787 (6)	0.4366 (6)	0.0508 (13)	

### Atomic displacement parameters (Å^2^)

**Table:**

	*U*^11^	*U*^22^	*U*^33^	*U*^12^	*U*^13^	*U*^23^
S1	0.0445 (7)	0.0429 (6)	0.0560 (7)	−0.0110 (4)	−0.0040 (5)	0.0114 (5)
S2	0.0417 (6)	0.0382 (6)	0.0480 (6)	−0.0064 (4)	0.0010 (5)	0.0072 (4)
S3	0.0439 (6)	0.0461 (6)	0.0408 (6)	0.0008 (5)	−0.0032 (5)	−0.0037 (4)
F1	0.059 (2)	0.108 (3)	0.078 (2)	−0.0333 (19)	0.0133 (17)	−0.009 (2)
F2	0.073 (3)	0.079 (2)	0.152 (4)	0.0122 (19)	0.008 (3)	−0.008 (2)
F3	0.090 (3)	0.071 (2)	0.125 (3)	−0.0072 (19)	0.005 (2)	−0.028 (2)
F4	0.094 (3)	0.311 (8)	0.073 (3)	−0.079 (4)	−0.022 (2)	0.071 (4)
O1	0.051 (2)	0.058 (2)	0.059 (2)	−0.0042 (15)	−0.0088 (16)	0.0206 (16)
O2	0.0385 (17)	0.061 (2)	0.0575 (19)	−0.0113 (15)	−0.0048 (15)	0.0072 (15)
C1	0.039 (2)	0.033 (2)	0.038 (2)	0.0008 (16)	0.0050 (17)	−0.0016 (15)
C2	0.042 (2)	0.042 (2)	0.044 (2)	−0.0021 (18)	0.0020 (18)	0.0048 (18)
C3	0.033 (2)	0.041 (2)	0.042 (2)	−0.0013 (16)	−0.0003 (17)	0.0028 (17)
C4	0.074 (5)	0.097 (4)	0.071 (5)	−0.025 (3)	−0.029 (4)	0.035 (3)
C4'	0.074 (5)	0.097 (4)	0.071 (5)	−0.025 (3)	−0.029 (4)	0.035 (3)
C5	0.074 (5)	0.097 (4)	0.071 (5)	−0.025 (3)	−0.029 (4)	0.035 (3)
C5'	0.074 (5)	0.097 (4)	0.071 (5)	−0.025 (3)	−0.029 (4)	0.035 (3)
C6	0.069 (3)	0.057 (3)	0.054 (3)	0.007 (3)	−0.007 (2)	0.013 (2)
B1	0.044 (3)	0.054 (3)	0.055 (3)	−0.005 (2)	0.003 (2)	−0.002 (2)

### Geometric parameters (Å, º)

**Table:**

S1—C1	1.691 (4)	O2—C5	1.450 (15)
S1—C2	1.723 (5)	C2—C3	1.341 (6)
S2—C1	1.681 (4)	C4—C5	1.493 (18)
S2—C3	1.723 (4)	C4—H4A	0.9800
S3—C1	1.717 (4)	C4—H4B	0.9800
S3—C6	1.797 (5)	C4'—C5'	1.456 (16)
F1—B1	1.379 (6)	C4'—H4'1	0.9800
F2—B1	1.373 (7)	C4'—H4'2	0.9800
F3—B1	1.392 (7)	C5—H5A	0.9800
F4—B1	1.332 (7)	C5—H5B	0.9800
O1—C2	1.356 (5)	C5'—H5'1	0.9800
O1—C4'	1.435 (13)	C5'—H5'2	0.9800
O1—C4	1.473 (14)	C6—H6A	0.9700
O2—C3	1.347 (5)	C6—H6B	0.9700
O2—C5'	1.427 (15)	C6—H6C	0.9700
			
C1—S1—C2	95.1 (2)	O1—C4'—H4'2	107.9
C1—S2—C3	95.4 (2)	C5'—C4'—H4'2	107.9
C1—S3—C6	101.0 (2)	H4'1—C4'—H4'2	107.2
C2—O1—C4'	115.0 (6)	O2—C5—C4	103.7 (14)
C2—O1—C4	104.8 (6)	O2—C5—H5A	111.0
C4'—O1—C4	18.0 (12)	C4—C5—H5A	111.0
C3—O2—C5'	113.3 (9)	O2—C5—H5B	111.0
C3—O2—C5	108.7 (9)	C4—C5—H5B	111.0
C5'—O2—C5	13 (2)	H5A—C5—H5B	109.0
S2—C1—S1	116.8 (3)	O2—C5'—C4'	121.7 (15)
S2—C1—S3	124.5 (2)	O2—C5'—H5'1	106.9
S1—C1—S3	118.8 (2)	C4'—C5'—H5'1	106.9
C3—C2—O1	125.3 (4)	O2—C5'—H5'2	106.9
C3—C2—S1	116.5 (3)	C4'—C5'—H5'2	106.9
O1—C2—S1	118.2 (3)	H5'1—C5'—H5'2	106.7
C2—C3—O2	125.0 (4)	S3—C6—H6A	109.5
C2—C3—S2	116.2 (3)	S3—C6—H6B	109.5
O2—C3—S2	118.6 (3)	H6A—C6—H6B	109.5
O1—C4—C5	110.2 (18)	S3—C6—H6C	109.5
O1—C4—H4A	109.6	H6A—C6—H6C	109.5
C5—C4—H4A	109.6	H6B—C6—H6C	109.5
O1—C4—H4B	109.6	F4—B1—F2	111.2 (5)
C5—C4—H4B	109.6	F4—B1—F1	111.2 (5)
H4A—C4—H4B	108.1	F2—B1—F1	111.7 (5)
O1—C4'—C5'	117.8 (13)	F4—B1—F3	108.9 (5)
O1—C4'—H4'1	107.9	F2—B1—F3	104.1 (5)
C5'—C4'—H4'1	107.9	F1—B1—F3	109.4 (4)
			
C3—S2—C1—S1	1.2 (3)	C5'—O2—C3—C2	−6.9 (13)
C3—S2—C1—S3	−178.9 (3)	C5—O2—C3—C2	−20.1 (11)
C2—S1—C1—S2	−0.6 (3)	C5'—O2—C3—S2	168.9 (12)
C2—S1—C1—S3	179.5 (3)	C5—O2—C3—S2	155.7 (10)
C6—S3—C1—S2	2.2 (3)	C1—S2—C3—C2	−1.7 (4)
C6—S3—C1—S1	−177.9 (3)	C1—S2—C3—O2	−177.8 (3)
C4'—O1—C2—C3	−0.6 (12)	C2—O1—C4—C5	53.2 (14)
C4—O1—C2—C3	−16.3 (10)	C4'—O1—C4—C5	−74 (5)
C4'—O1—C2—S1	−178.1 (11)	C2—O1—C4'—C5'	8 (3)
C4—O1—C2—S1	166.1 (8)	C4—O1—C4'—C5'	67 (4)
C1—S1—C2—C3	−0.6 (4)	C3—O2—C5—C4	53.0 (19)
C1—S1—C2—O1	177.2 (4)	C5'—O2—C5—C4	−60 (6)
O1—C2—C3—O2	−0.1 (7)	O1—C4—C5—O2	−75 (2)
S1—C2—C3—O2	177.4 (3)	C3—O2—C5'—C4'	15 (3)
O1—C2—C3—S2	−176.0 (4)	C5—O2—C5'—C4'	87 (7)
S1—C2—C3—S2	1.5 (5)	O1—C4'—C5'—O2	−16 (4)
